# Employer branding, organization’s image and reputation, and intention to apply: the moderating role of the availability of organizational information on social media

**DOI:** 10.3389/fsoc.2024.1256733

**Published:** 2024-04-08

**Authors:** Nguyen Ngoc Thang, Pham Thu Trang

**Affiliations:** ^1^Hanoi School of Business and Management, Vietnam National University, Hanoi, Vietnam; ^2^IPAG Business School, Paris, France

**Keywords:** employer branding, social media, organization’s image and reputation, Generation Z, job seeker’s application intention

## Abstract

The topics of employer branding and organization’s image and reputation have been well-researched in the literature. However, most empirical studies were conducted in Europe, Australia, or the United States, but very few were conducted in Asia, especially in Vietnam. In addition, the interaction of image and reputation with the availability of information on social media is poorly understood. Using signaling theory for building a research model, we collected data from 206 Generation Z respondents from the logistics sector in Vietnam. Our findings show that (i) employer branding has positively and significantly related to an organization’s image and reputation; (ii) the organization’s image and reputation had a significant effect on job seekers’ intention to apply; and (iii) the interaction of image and reputation with availability of information on social media to predict the job seeker’s intention to apply. The paper also presented implications for both researchers and practitioners as well as recommendations for future research.

## Introduction

In the modern business environment, a company’s ability to attract top talent depends on its reputation and image. In fact, there has been much research focused on employer branding and talent acquisition (Lievens, 2007; Collins and Kanar, 2014; Lievens and Slaughter, 2016), however, with the rapidly changing technology, the present economy is connected globally and virtually through a different range of sources in both informal and formal forms such as social media, networking platforms or websites. Employer branding is more difficult since workplace information is becoming commonplace via social media platforms (McFarland and Ployhart, 2015). In a highly competitive marketplace, as competition for customers, companies need to complete and attract the best workers (Berthon et al., 2005). Thus, companies have to differentiate themselves from their competitors and build their reputation and image to retain current employees and attract prospective applicants (Lievens and Highhouse, 2003). Accordingly, several companies have developed standard and official employer branding programs, in which they have spent considerable resources on campaigns, advertisements, websites, and social networks. In addition, companies need also to understand the job seekers’ expectations if companies want to recruit them.

Furthermore, the modern business environment is characterized by cross-border competition, technical advancements, the expansion of the intellectual economy, and the need for flexibility and knowledge in the workplace (Ahi et al., 2022). Besides, due to challenges from a shifting demographic base and an increased need for talented people, there is a greater demand than there is supply for highly competent and motivated applicants (Ployhart, 2006). However, the global movement and international interaction also allow employers to attract more qualified and talented applicants to obtain more excellent utility in their selection systems. Studies on the link between the use of social media and organization attractiveness have been carried out in the United Kingdom, Australia, India, and Turkey (Berthon et al., 2005; Knox and Freeman, 2006; Srivastava and Bhatnagar, 2010; Alnıaçık et al., 2014), but they may different from Vietnamese studies in terms of national aspects (Nguyen et al., 2018). In Vietnam, the elements that compose attractive employer branding may differ from other parts of the work. As Vietnam is a young and dynamic developing economy, multinational corporations should have an insight into the applicant pool in Vietnam to develop their appropriate employee value proposition (Srivastava and Bhatnagar, 2010; Thang et al., 2022a,b).

Social media has developed enormously due to the growth of the internet under a wide range of platforms. Naturally, corporations take advantage of these channels to expand their image and reputation. For example, 94 percent of the world’s fastest-growing companies use LinkedIn to recruit employees (Barnes et al., 2015). Even though there are pros and cons of using social networks for employer branding and recruitment, it is undeniable that platforms such as Facebook, with over one billion users, have great potential. First of all, with audio-visual tools, social media tends to be more attractive than traditional media channels. Previous research has shown that instrumental and symbolic employer image dimensions affect potential applicants’ attractiveness perceptions (Lievens and Highhouse, 2003; Van Hoye and Saks, 2011). Secondly, information in these channels is not one-way perceived but evaluated and discussed by audiences. With the interaction among audiences, both recruiter and job seeker can be beneficial as the former can understand the need to provide more information, and the latter can search, evaluate and select the most reliable and suitable recruiter for themselves. Job seekers can verify the information and image that one company provides on social networks, so it is more likely that employees use social network channels for collecting information, but little is test about the processes. Thus, the research of employer branding on social media on perceived employer attractiveness, organization’s image and reputation and employee’s intention to apply in Vietnam is urgent and important. From the paper, we can have a deeper look at a candidate’s intention to apply due to this factor. The findings from this study shall contribute to the literature on the use of social media and employer branding and the field of human resource and recruitment.

## Employer branding on social media and recruitment

### Employer branding and employer image

Ambler and Barrow (1996) defined employer branding encompasses the practical, financial, and psychological benefits that an organization might offer to job seekers. This definition is exclusive to each organization and aids in differentiating them from one another. In other words, organizations adopting particular strategies require certain types of benefits that will differ from those required by organizations with different strategies. Each organization may increase the effectiveness of recruiting and retaining staff by investing in employer branding (Backhaus and Tikoo, 2004). Other researchers (e.g., Minchington, 2006) suggested that employer branding can be viewed as the organization’s image established by the Directors Board in order to become the ideal workplace for employees. Additionally, employer branding initiatives entice additional external potential employees to work for the business. These initiatives center on improving the organization’s reputation as a potential employer in the labor market (Lievens and Slaughter, 2016). From an interdisciplinary approach, some researchers (e.g., Maheshwari et al., 2017) argued that employer branding is the idea at the confluence of the marketing and human resources fields because building an image in the minds of the potential labor market is a marketing activity in order to attract and retain employees for organizations. At present, employer branding has become an important tool for organization to enhance organization’s image and reputation and acquire prospective job seekers and retain present employees (Junça Silva and Dias, 2023).

Companies can use employer branding in two different directions. Internal branding is directed at the employer’s image of the organization, the communication among employees, and how employees interpret it. In contrast, external branding relates to how the organization exposes itself and how employers evaluate it (Sivertzen et al., 2013). Employer branding makes a company more appealing and enhances its reputation (Chapman et al., 2005; Jones et al., 2014). Employer attraction is characterized as the anticipated advantages that potential employees see in working for a certain company. The construct might be the more specific idea of employer brand equity. Significantly, an organization’s employer brand fairness is strengthened the more favorably prospective workers view it (Thang et al., 2022b). The organization will appeal to a potential employee if their needs, personalities, and values match those of the organization (Lievens and Slaughter, 2016).

From a job seeker’s perspective, employer branding has been considered as the perceived benefits of a position in an organization. According to Berthon et al. (2005), employer branding included five dimensions: (i) interest value; (ii) social value; (iii) economic value; (iv) development value; and (v) application value. Firstly, the interest value of a product or service is related to innovation and customer interest. Secondly, the social value emphasizes the working environment and interactions among coworkers. Thirdly, the economic value includes benefits, compensation and rewards. Fourthly, the development value indicates the potential for future employment prospects. Lastly, application value demonstrates how customer-focused an organization is and includes the potential to apply prior knowledge. Therefore, it is important for an organization to convert its benefits into value propositions that job seekers can understand and convey with ease. If there is a lack of effort to integrate these benefits into the organization’s image and reputation, job seekers will not know about the organization. In addition, the organization will not be recognized without these organizational communication initiatives. Accordingly, we propose the following hypotheses:

*Hypothesis 1*: The job seeker's perception of the five dimensions of employer branding positively affects the organization's image and reputation.

*Hypothesis 1a*: The job seeker's perception of the interest value of employer branding positively affects the organization's image and reputation.

*Hypothesis 1b*: The job seeker's perception of the social value of employer branding positively affects the organization's image and reputation.

*Hypothesis 1c*: The job seeker's perception of the economic value of employer branding positively affects the organization's image and reputation.

*Hypothesis 1d*: The job seeker's perception of the development value of employer branding positively affects the organization's image and reputation.

*Hypothesis 1e*: The job seeker's perception of the application value of employer branding positively affects the organization's image and reputation.

### Employer image and reputation on social media and recruitment

#### The universal approach to employer image and reputation on social media

The interest in employer image resulting from employer branding has drawn the attention of researchers in human resource practices. Researchers believe that job seekers’ impressions of a company’s image may affect their interest in it as a potential employer. There is a wave of studies in order to provide a better comprehending employees’ perceptions of their employer’s image and reputation (Sivertzen et al., 2013; Thang et al., 2022b). However, in a critical review of the factors affecting corporate image and organizational identity, Lievens and Slaughter (2016) concluded that organizational attractiveness depends not only on the functional aspects of a job but also on the symbolic value attached to working for a particular company. They also suggested that symbolic elements were related to people’s desires to maintain their sense of self, improve their self-image, or express themselves. Job seekers tended to be drawn to apply for companies that shared their characteristics. These symbolic qualities give a subjective and intangible description of the position or organization, and they may be important in drawing young people to it via social media and websites (Robertson et al., 2019; Dassler et al., 2022).

One of the most widely used paradigms in recruitment research is signaling theory, which is usually used to explain how organizations can affect prospective applicants’ impressions of an employer (Chapman et al., 2005). In order to decide about whether to apply for a job from an organization, job seekers need to know about the organization. However, they usually lack comprehensive information about what it is like to work there. As a result, anything job seekers read, hear, or see about a prospective employer is taken to be conveying signals about the organization’s characteristics (Uggerslev et al., 2012; Carpentier et al., 2019). Thus, social networks are currently accepted and widely used as a tool for employer branding due to their many advantages, including their free, limitless use, major interaction, and quick reaction times (Ollington et al., 2013; McDonald et al., 2022). Recent research also suggested that the trust that younger job seekers have in the information published on social media and the Internet has a significant impact on their decision to apply (Thang et al., 2022a). In today’s digital world, social media platforms are considered as one of the key communication channels and even employers can proactively provide information about their organization and job opportunities on social media pages (Laukkarinen, 2023). Accordingly, they suggest:

*Hypothesis 2*: The organization's image and reputation positively affect the job seeker's intention to apply.

Besides traditional advertising channels such as newspapers or websites, the internet has opened more opportunities for advertising and sharing information through social networks such as Facebook, Instagram, Tiktok, LinkedIn (Haenlein et al., 2020). Social networks allow employers to find and evaluate candidates and attract active and passive job seekers (Laukkarinen, 2023). One thing unique about social networks is that users can make public profiles online, and others can visit them. This helps extend the network and connection, giving active candidates opportunities to find a job and helping recruiters find both active and passive job seekers. It is widely claimed that social networking channels are allegedly more effective at attracting passive applicants quickly and at higher conversion rates (Suen, 2018).

In the past, both active and passive information asymmetries have resulted from historically quite strict control over the information and image that employers and job searchers portray to one another (Bangerter et al., 2012; Sivertzen et al., 2013). However, the social media platform landscape has changed quickly through various technologies and networks during the last decade. Social media applications have become incredibly popular among internet users, which has forced organizations to modify their hiring practices and embrace social media as a tool for hiring. These changes have also affected how recruiters and job seekers use social media to find professionally relevant content (Kane, 2017). Thus, organizations are now making organizational information available on social media and keeping it there in order to increase their salience on social networks, cultivate an online community, and spark interest in their prospective employees. This argument yields the following hypothesis:

*Hypothesis 3*: The job seeker's perception of the availability of organizational information on social media positively affects the job seeker's intention to apply.

#### A contingency approach to employer image and reputation on social media

Recruitment literature shows that job seekers take into consideration any information about the characteristics of the organization and are swayed by it. More specifically, job seekers can find information through phone calls, interviews, or word-of-mouth at the individual level, while information at the organizational level, on the other hand, includes details from job postings and vacancy advertisements. Studies have claimed that a job seeker interprets various activities connected to the recruitment process and employer information as indicators of organizational characteristics (Thang et al., 2022b). Lack of information might lead to the job seeker forming an inaccurate or incomplete impression based on signals that are communicated to them and their own interpretations (Younis and Hammad, 2021). In terms of signals forming the corporate image, organizations should implement branding strategies that work to create favorable perceptions of them as a distinctive and desirable place to work. In order to be appealing and establish a positive reputation as an employer, businesses must take into account the context of the information provided to stakeholders, the information sources, and the reliability of each source (Lievens and Slaughter, 2016; Kashive and Khanna, 2017).

According to the contingency approach, the impact of an organization’s image and reputation on job seekers’ intention to apply for a job is conditioned by the availability of organizational information on social media (Sivertzen et al., 2013; Younis and Hammad, 2021). For instance, an organization’s image and reputation would be more likely to influence job seekers’ intention to apply for a job if the firm’s recruitment strategy relies on or utilizes the availability of organizational information on social media; otherwise, the connection between an organization’s image and reputation and job seekers’ intention to apply for a job might be weak. Indeed, to create correct perceptions of the employer, the job seeker is motivated to find out as much information as possible about the firm or the open position. They may determine whether or not it is a good place to apply for jobs by consulting the information that the organization is ready to provide (Myrden and Kelloway, 2015). Therefore, we propose the following hypothesis:

*Hypothesis 4*: The job seeker’s perception of the availability of organizational information on social media positively moderates the relationship between an organization's image and reputation and job seekers’ intention to apply for a job.

**Figure 1:**
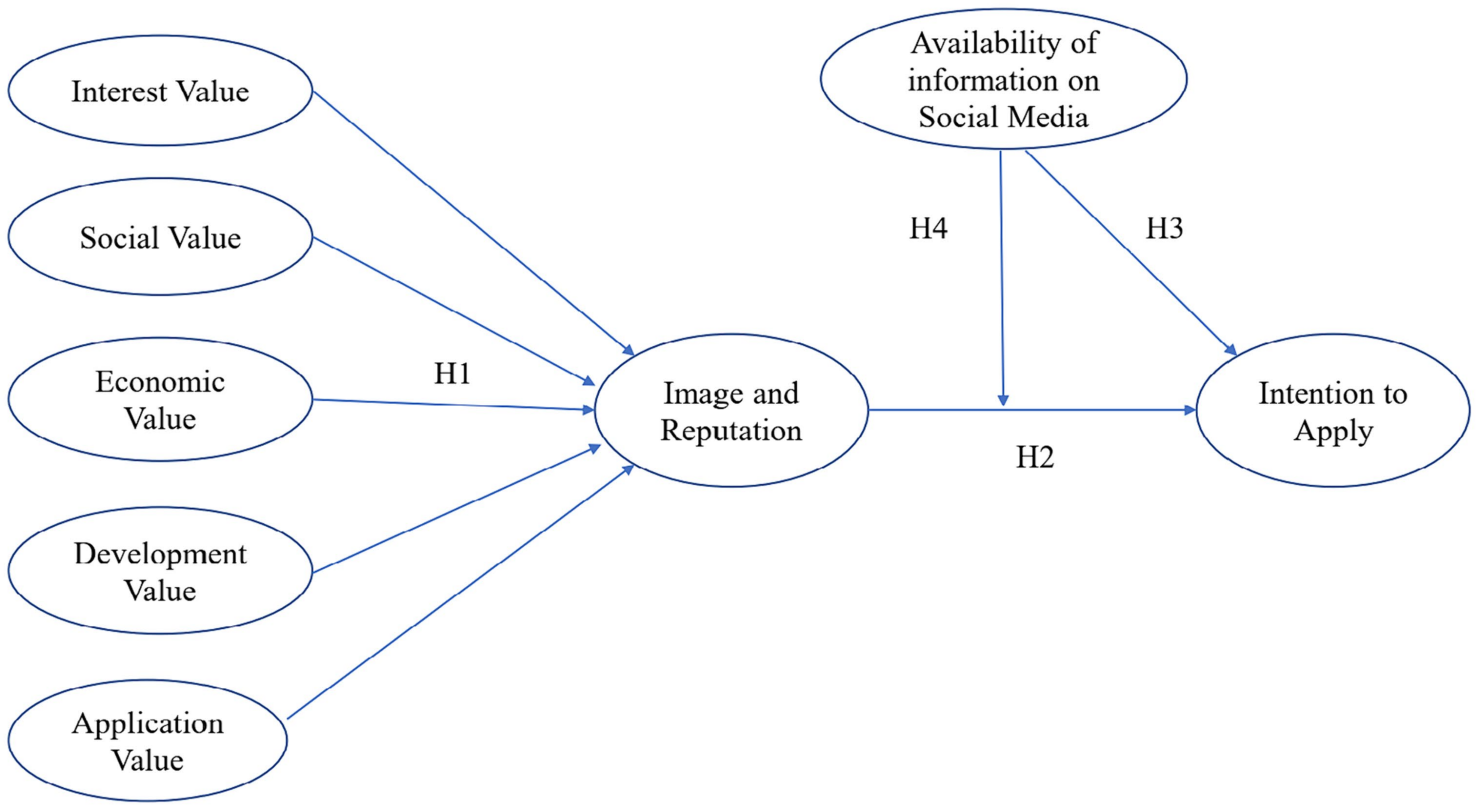
Research model.

In summary, based on the above hypotheses that link employer branding, availability of information on social media, organization’s image and reputation, and intention to apply, we proposed our research model as shown in Figure 1.

## Methodology

### Data and procedures

A web-based survey was applied because of the difficulty in physical contact due to the COVID-19 pandemic. In May 2022, 500 questionnaires were sent out to final-year students belonging to the logistics major in Vietnam and 206 usable questionnaires were collected. The response rate was 55% and considered acceptable because other studies in the same research area by Dumont et al. (2017), Tang et al. (2018), and Obeidat et al. (2020) showed that response rates are 60, 53, and 48%, respectively. Final-year students are the perfect subject of this research as they are prospective job seekers. They are suitable for this study because they are Generation Z, born in the 1997–2012 period, the primary social media users. Seventy-six percent of Generation Z users say that social media enables them to interact with brands and companies, and 78% report using social media to learn about new brands (Nielsen, 2012). Thus, companies who invest in employer branding shall have a more significant influence on this generation. We conducted the study with final-year undergraduate students studying in logistics-related majors, asking them questions about their opinions on five top logistics companies in Vietnam that have a wide network of nationwide service centers. In addition, these companies all have advertising on the internet and social media; hence, it shall provide good examples for participants to give opinions.

### Measures

Employer attractiveness was measured using Berthon et al. (2005) 25-items. Employer attractiveness scale included dimensions, such as interest value (e.g., “The organization both values and makes use of your creativity”), development value (e.g., “Feeling more self-confident as a result of working for a particular organization”), social value (e.g., “Supportive and encouraging colleagues”), economic value (e.g., “An above-average basic salary”), and application value (e.g., “Opportunity to teach others what you have learned”). A five-point Likert scale with a range of 1 (to a very small extent) to 5 (to a very great extent) was used to measure these aspects.

The intention to apply for a job was measured with four items adapted from Lam (2007). For example, “I am very interested in pursuing my application with this company if offered one,” or “I feel I know enough about this company to no longer be interested in it “. A five-point Likert scale - from 1 (not at all accurate) to 5 (extremely accurate) – was used to score the items.

The availability of organizational information on social media was measured with four items adapted from Collins and Kanar (2014). The scale was used for marketing of organizations in traditional methods such as flyers, brochures, and school newspapers, but in this study, we modified the scale to apply to the usage of social media. A sample item like “I have seen advertising for jobs at this organization in social media.” A Likert scale with five possible outcomes – from 1 (not at all accurate) to 5 (extremely accurate) – was used to evaluate the items.

The organization’s image and reputation are calculated based on scale by Turban et al. (1998) including four items like “I have heard many good things about this company” or “I have never heard about this company.” A five-point Likert scale – from 1 (not at all accurate) to 5 (extremely accurate) - was used to score the items.

Some control variables were also asked in the survey. These are gender, age, living location, study major, and work experience. Data from demographic questions were discussed using descriptive analysis, while Likert questions were tested for reliability, validity, and discrimination validity. In addition, bootstrapping analysis was used to examine the statistical significance of various PLS-SEM outcomes in path coefficients, Cronbach’s alpha, HTMT, and *R*^2^ values.

## Results

### Demographic characteristics

According to the survey, 70.07% of the participants were female, while males accounted for 29.93%. In terms of age, more than 32% of the respondents were 20, 19.71% were 21 years old, 18.89% were 22 years old, and less than 6% were 23–25 years old. More than half of the participants have not had any job experience (53.28%), while the remaining have done an internship or had some job experience. Regarding location, even if 100% of them studied at Universities around Hanoi, they conducted an online study when the survey was taken. Their living location is various, but most of them (71.53%) lived in Hanoi, followed by Ho Chi Minh City with 7.30%, and other locations were less than 4%.

### Data analysis

PLS-SEM was utilized in this work to analyze the data. This approach is helpful when predicting dependent variables from a wide range of independent factors. With the help of SEM, and multivariate statistical methods, we can estimate and investigate causal links between dependent variables and independent variables. The sorts of variables in these models are separated based on how they are measured or how they are used in the model.

### Outer loadings

The main indicators of the trajectory of the latent variable toward the observable variables in reflective models are the outer loadings. They demonstrate the extent to which each observable variable or item contributes absolutely to the latent variable or construct description. According to Hulland (1999), loadings are typically anticipated to be more than 0.7. Thus, we have eliminated all items that have loadings lower than 0.7.

### Assessment of reliability and validity

The primary objective of the questionnaire when conducting research is to gather pertinent, accurate, and legitimate information. Consequently, the consistency and accuracy of a questionnaire form a crucial aspect of a research methodology known as reliability and validity (Cooper and Schindler, 2006).

Regarding the participants’ responses, all scales in Table 1 have Cronbach’s Alpha values higher than 0.7, indicating this scale has an appropriate level of internal consistency. Reliability is assessed using Composite Reliability in addition to Cronbach’s Alpha. Furthermore, the Composite Reliability of all scales is more significant than 0.7. Therefore, according to the Composite Reliability and Cronbach’s Alpha tests, all scales are valid.

**Table 1:** Cronbach’s alpha and composite reliability.

Variables	Cronbach’s alpha	Composite reliability
Interest value	0.836	0.891
Social value	0.814	0.877
Economic value	0.848	0.897
Development value	0.872	0.913
Application value	0.887	0.917
Intention to apply	0.857	0.904
Image and reputation	0.875	0.915
Availability of information on social media	0.818	0.880

A scale is said to have achieved convergence, according to Hock and Ringle (2010), if the average variance index is 0.5 or greater. The Average Variance Extracted is all more than 0.5 after evaluating the indicators’ dependability and validity, assuring convergence validity. Table 2 shows that the Average Variance Extracted are all greater than 0.5, ensuring convergence validity.

**Table 2:** Average variance extracted (AVE).

Variables	Average variance extracted (AVE)
Interest value	0.671
Social value	0.641
Economic value	0.686
Development value	0.723
Application value	0.689
Intention to apply	0.701
Image and reputation	0.728
Availability of information on social media	0.647

### Model fit test

To assess model fit, confirmatory factor analysis was then employed. Model fit values are shown in Table 1. According to Hair et al. (2010), values of Chi-square/df less than 2 and RMSEA less than 0.08 indicates a good model fit. In the study, the Chi-square/df and RMSEA values are 1.278 and 0.037, respectively, indicating evidence of good fit. Furthermore, values of CFI and TLI are close to scoring 1 which can indicate well-fitting model. Other value, such as PCLOSE, also suggest a good model fit. Overall, model fit values suggest that the model has a good fit for the data in our sample (Table 3).

**Table 3:** Overall model goodness of fit.

No.	Indicators	Result
1	Chi-square/df	1.278
2	GFI	0.856
3	CFI	0.964
4	TLI	0.959
5	RMSEA	0.037
6	PCLOSE	0.996

With the HTMT ratios, according to Garson (2016), the HTMT ratios ensure the discriminant value between the two latent variables when it is smaller than 1. Table 4 shows that all HTMT ratios are smaller than 0.9, guaranteeing the discriminability validity.

**Table 4:** Heterotrait-monotrait ratio (HTMT).

Variables	1	2	3	4	5	6	7	8
1. Application value	1							
2. Development value	0.470	1						
3. Economic value	0.325	0.312	1					
4. Intention to apply	0.548	0.547	0.401	1				
5. Image and reputation	0.686	0.645	0.560	0.892	1			
6. Interest value	0.277	0.180	0.138	0.287	0.509	1		
7. Availability of information on social media	0.204	0.146	0.124	0.126	0.233	0.067	1	
8. Social value	0.530	0.348	0.256	0.498	0.678	0.367	0.201	1

### Hypotheses testing

According to Hair et al. (2006), the model has a very high likelihood of exhibiting multicollinearity if the VIF is 5 or higher. Our results show that both inner and outer VIF values are less than 5, so there is little chance that multicollinearity occurred. In this study, four hypotheses were proposed to be tested by conducting a web-based survey for final-year and freshly graduated students who majored in the logistics-related field at a university in Vietnam. Four hypotheses were about the relation between employer branding (including five dimensions), the organization’s image and reputation, the availability of the organization’s information on social media, and the job seeker’s intention to apply. SPSS 24 was used to measure these relations, and the results are presented in Table 5.

**Table 5:** Path coefficients.

Path as represented in Figure 1	Estimate
Interest value → Image and reputation	0.224^**^
Social value → Image and reputation	0.258^**^
Economic value → Image and reputation	0.257^***^
Development value → Image and reputation	0.277^**^
Application value → Image and reputation	0.239^**^
Image and reputation → Intention to apply	0.767^**^
Availability of information on social media → Intention to apply	−0.040
Availability of information on social media x image and reputation → intention to apply	0.125^*^

*N* = 206, in this study, gender, age, and work experience were controlled. ^*^p < 0.05, ^**^p < 0.01.

Table 5 reveals the findings for all hypotheses. The results show that all *p*-value of the relation between five dimensions of employer branding, including application value, development value, economic value, social value, and interest value and the organization’s image and reputation are lower than 0.01. These findings suggest that five dimensions of employer branding were positively and significantly related to the organization’s image and reputation. Overall, these findings provide preliminary support for Hypothesis 1. Thus, the results implied that the better the employer branding, the greater the image and reputation of these organizations. Additionally, the development value has the highest impact on an organization’s image and reputation among the five dimensions of employer branding, while interest value has the weakest impact on an organization’s image and reputation.

It can be seen from Table 5 that the organization’s image and reputation positively affect job seekers’ intention to apply because the standardized path coefficient for the organization’s image and reputation on job seekers’ intention to apply is positively significant (*b* = 0.767, *p* < 0.01). This result provides support for Hypothesis 2 and suggests that an organization’s good image and reputation is a valuable approach for attracting Generation Z job seekers who are studying logistics-related majors.

With gender, age, living location, study major, and work experience controlled, Table 5 shows that the *p*-value of the relation between the availability of organizational information on social media and the job seeker’s intention to apply is higher than 0.05. Thus, this result provides no support for Hypothesis 3.

Beyond the direct relationships between (i) employer branding and the organization’s image and reputation, and (ii) the organization’s image and reputation and the job seeker’s intention to apply, we also found support for the contingency approach to employer image and reputation on social media. As a set, image and reputation - availability of information on social media interaction terms had significant effects on the job seeker’s intention to apply, thereby providing support for Hypothesis 4. These moderation results provide strong evidence that the availability of information on social media influences the relationship between the organization’s good image and reputation and the job seeker’s intention to apply. In short, maximizing a job seeker’s intention to apply appears to depend on properly aligning image and reputation with the availability of information on social media.

## Discussion

The purpose of this study is to investigate the link between five dimensions of employer branding, the organization’s image and reputation, the availability of the organization’s information on social media, and the job seeker’s intention to apply. The study contributes to the literature by developing and testing signaling theory regarding how and why employer branding, the availability of the organization’s information on social media and organization’s image and reputation are attractive to job seekers. Our findings reveal that employer branding has a positive effect on the organization’s image and reputation, while the availability of the organization’s information on social media was a key moderating influence on the relationship between organization’s image and reputation and the job seeker’s intention to apply of our sample. From the regression analysis, it can be identified that three Hypotheses 1, 2, and 4 were supported, while Hypothesis 3 was not confirmed. The research findings will be discussed as follows.

First, as expected in the Hypothesis 1 we found that there is a positive association between five dimensions of the employer branding and the organization’s image and reputation. While this idea has been clearly stated in the literature on recruitment (e.g., Younis and Hammad, 2021), our findings shed light on the specific aspects of employer branding influence organization’s image and reputation. Specifically, the dimension of development value had the highest positive impact on the organization’s image and reputation, while interest value had the lowest effect on the organization’s image and reputation. This finding is significantly helpful for an employer as it identifies and ranks the employer branding attractiveness indicators. Such findings are consistent with Junça Silva and Dias (2023), who found that all aspects of employer branding were linked to candidates’ attraction to jobs and had an impact on their choice. It appears that job seekers must assess the possible benefits offered by the organization before deciding whether or not to think highly of it as an employer, particularly for final-year students who may lack information about employer benefits and characteristics. Contrary to our findings, the study of Sivertzen et al. (2013) did not find support for the links between social value and economic value with corporate reputation. We speculated that this has resulted from the difference in research contexts in developing countries and developed countries because job seekers in developing countries are often more concerned about economic value than job seekers in developed countries.

Second, the positive relation of an organization’s image and reputation with job seekers’ intention to apply indicates that investing in building a good firm image may result in higher job applications. The potential candidates who positively perceive the organization’s image and reputation may believe that they will feel better and more likely to apply for that company. Previous studies show a connection between the organization’s image and reputation and job pursuit intentions (Jones et al., 2014; Chaudhary, 2019). Our results indicate that organization’s image and reputation in fact had a significant effect the job seeker’s intention to apply. Such findings coincide with the previous studies (e.g., Carpentier et al., 2019; Junça Silva and Dias, 2023) that promote the use of employer branding and organization’s image and reputation as a means to foster recruitment. Even though these studies offer valuable insights, most job searchers may lack sufficient knowledge about the prospective employer. A barrier that restricts the impact of an organization’s image and reputation on recruitment outcomes is the fact that people are occasionally largely unaware of an organization’s image and reputation. Thus, our study extended previous studies and our understanding of the relationship among of employer branding, organization’s image and reputation, and intention to apply by investigating how to use social media in creating an organization’s image and reputation and employer attractiveness to job seekers. This is why our hypotheses were proposed to test whether there is any relation between the availability of information on social media and other aspects.

Third, besides the direct relationships between an organization’s image and reputation and job seekers’ intention to apply, our result indicates that the availability of information on social media does in fact moderate the relationship between an organization’s image and reputation, and job seekers’ intention to apply. This is an interesting finding because not all previous studies (e.g., Sivertzen et al., 2013) was able to support the relationship. It can be explained as the subject of this study – Generation Z is exposed to social media that they gain more reliability and trust for the information available on this platform than former generations (Thang et al., 2022b). From our findings, it appears that organizations are capitalizing on the opportunity to improve recruitment by combining the organization’s image and reputation with the availability of information of the organization on social media because social media plays an important role in labor market by providing a low-cost means of communication between organizations and job seekers regarding employment-related information. Thus, our study contributes to recruitment literature and extends signaling theory by using the availability of information on social media as a moderator factor in investigating the relationships between an organization’s image and reputation, and job seekers’ intention to apply.

Finally, our results indicate that there was no connection between the availability of information on social media and the job seeker’s intention to apply. This can be explained as the availability of information can be provided through various channels and methods such as word-of-mouth, television, newspaper, flyer. Similarly, not all company with an enormous amount of information on social media has information that job seekers need or are interested in during the job search process. Furthermore, it appears that organizations were not making a consistent connection between the social media strategy they pursued and the recruitment activities they employed.

## Conclusion

In the digital society, the use of social media to enhance employer branding and an organization’s image and reputation is receiving a good deal of emphasis. Our findings show that (i) five dimensions of employer branding have positively and significantly related to the organization’s image and reputation; (ii) the organization’s image and reputation had a significant effect on job seekers’ intention to apply; and (iii) the interaction of image and reputation with availability of information on social media to predict the job seeker’s intention to apply. These findings indicate that organizations should concentrate on building their employer brands and recruiting their prospective employees through the development of social and economic aspects. These aspects may include recognition from the manager, experience and future opportunities in career path, self-confidence and self-satisfaction feeling while working in the company, good relationships, a healthy working environment, an attractive compensation package, the opportunity for promotion, and job security. These elements are crucial for establishing a positive company reputation, which will increase potential employees’ intent to apply for jobs. This positive relationship can also be affected by providing an adequate amount of the company’s information on social media. Thus, businesses should not only develop official employer branding programs but also pay more attention to the availability of information on company websites and social media, recruitment campaigns, and advertisements if they would like to attract prospective applicants. However, our research has some limitations that provide opportunities for future research. First, it was carried out in a fabricated environment with a convenience sample. Using probability sampling from larger populations might provide future studies with external validity. Additionally, because of the cross-sectional nature of this study, causal implications cannot be drawn. Future research may apply confirmatory factor analysis to investigate the construct validity of the employer attractiveness survey. This effort will aid in changing the scale to fit a different situation. Second, logistics students, who are in high demand in the labor market, were selected to participate in our survey. Thus, businesses compete with one another to hire the best candidates, and employer branding is a key topic in this circumstance. There are variations between industries. In addition, the use of student responses generally has both advantages and disadvantages. More specifically, it may impair external validity and limit the scope for generalization. Thus, future research needs to select other student majors or experienced job seekers in order to address existing concerns of sample size.

## Data availability statement

The raw data supporting the conclusions of this article will be made available by the authors, without undue reservation.

## Ethics statement

Ethical approval was not required for the study involving human participants in accordance with the local legislation and institutional requirements. Written informed consent to participate in this study was not required from the participants in accordance with the national legislation and the institutional requirements.

## Author contributions

PT: Conceptualization, Data curation, Investigation, Methodology, Writing – original draft, Writing – review & editing. NT: Writing – original draft, Writing – review & editing.
